# Predictive Values of Systemic Immune-Inflammation Index in New-Onset Atrial Fibrillation Following Coronary Artery Bypass Grafting

**DOI:** 10.21470/1678-9741-2021-0278

**Published:** 2023

**Authors:** Yucel Yilmaz, Saban Kelesoglu, Deniz Elcik, Rifat Ozmen, Nihat Kalay

**Affiliations:** 1 Department of Cardiology, Ministry of Health, Kayseri City Hospital, Kayseri, Turkey.; 2 Department of Cardiology, Faculty of Medicine, Erciyes University, Kayseri, Turkey.; 3 Department of Cardiovascular Surgery, Faculty of Medicine, Erciyes University, Kayseri, Turkey.

**Keywords:** Inflammation, Atrial Fibrillation, Coronary Artery Bypass, Reference Parameters, Sensitivity and Specificity

## Abstract

**Introduction:**

We investigated the relationship between the newly-defined systemic immune-inflammation index and the new-onset atrial fibrillation in patients undergoing coronary artery bypass grafting.

**Method:**

This study included 392 patients who underwent coronary artery bypass grafting. We divided the participants into two groups as those with and without new-onset atrial fibrillation. Prior to coronary artery bypass grafting, we evaluated blood samples, including systemic immune-inflammation index, and other laboratory parameters of the patients. We formulized the systemic immune-inflammation index score as platelet × neutrophil/lymphocyte counts.

**Results:**

The findings revealed that new-onset atrial fibrillation occurred in 80 (20.4%) of 392 patients during follow-ups. Such patients had higher systemic immune-inflammation index, neutrophil/lymphocyte ratio, and C-reactive protein levels than those who did not develop new-onset atrial fibrillation (P<0.001, P<0.001, P=0.010, respectively). In receiver operating characteristic curve analysis, systemic immune-inflammation index levels > 712.8 predicted new-onset atrial fibrillation with a sensitivity of 85% and a specificity of 61.2% (area under the curve: 0.781, 95% confidence interval: 0.727-0.835; P<0.001).

**Conclusion:**

Overall, systemic immune-inflammation index, a novel inflammatory marker, may be used as a decisive marker to predict the development of atrial fibrillation following coronary artery bypass grafting.

## INTRODUCTION

New-onset atrial fibrillation (NOAF) is the most prevalent arrhythmic complication in patients undergoing coronary artery bypass grafting (CABG). While the prevalence of atrial fibrillation (AF) in the general population is 1-2%, the frequency of NOAF development following CABG is 25-40%. Parallel to the improvements in surgery, the prevalence of NOAF development increases even more due to the advanced ages of the operated patients^[1]^. Patients with postoperative NOAF have an extended hospitalization and an increased risk of developing various complications such as cardiac events, kidney failure, infection, and cerebral infarction^[2]^.

It is suggested that many pathophysiological factors during the operation, such as myocardial damage and ischemia, catecholamine discharge, and oxidative stress, cause postoperative NOAF development^[3,4]^. However, the underlying reason behind the significant differences in NOAF development in some patients, despite having similar risk factors, is still unknown. After all, many studies report a strong and independent association between the increase in inflammation and various inflammatory markers and NOAF development after CABG^[5,6]^. Nevertheless, NOAF development following CABG procedures continues to be a severe complication. Therefore, early risk prediction for postoperative NOAF development before the operation is critical, and new biomarkers are needed to predict it.

Scholars previously showed that the systemic immune-inflammation index (SII), a recently introduced inflammation parameter, can be a strong prognostic indicator of adverse outcomes in various cancer types^[7]^. Previous studies suggest that biomarkers, such as neutrophil/lymphocyte ratio (NLR) and platelet/lymphocyte ratio (PLR) — relying on neutrophil, lymphocyte, and platelet counts —, can be used as prognostic indicators in various cardiovascular diseases, as well as for NOAF after CABG^[8]^. However, there is no previous study on the relationship between SII levels after CABG and NOAF development. Hence, in this study, we investigated the relationship between SII and NOAF development in patients after CABG, and, to the best of our knowledge, this was the first study exploring such a relationship.

## METHODS

### Study Population

This was a single-center, retrospective, and cross-sectional study conducted in our hospital. It included 392 patients who underwent CABG at Erciyes University Cardiovascular Hospital between March 2015 and September 2020. We recorded the basic clinical features, preoperative treatment modality, echocardiographic and angiographic findings, and intraoperative and postoperative parameters. The European System for Cardiac Operative Risk Evaluation (or EuroSCORE) II was calculated for each patient before CABG.

As in the medical data of the patients, postoperative control echocardiography was performed on all patients. Antiaggregant treatments were discontinued before the surgery. Similar procedures and standard solutions were used for the patients during the operations. Those with similar surgical techniques were included in the study. The study excluded patients with advanced left ventricular dysfunction (left ventricular ejection fraction (LVEF) < 30%), history of previous heart surgery and/or AF or utilizing antiarrhythmic therapy, younger age (< 18 years), hyperthyroidism, having undergone surgery without pumps, requirement of an intra-aortic balloon pump pre/peri/postoperatively, severe heart failure (New York Heart Association functional class III or IV), left atrial diameter > 55 mm, any inflammatory disease, urgent CABG, and pericarditis.

The study was approved by the institution’s human research committee (2020/642). We obtained informed consent from each patient and conducted the study protocols in accordance with the ethical guidelines of the 1975 Declaration of Helsinki.

### Laboratory Analyses

Antecubital venous blood samples of all patients were taken into tripotassium ethylenediaminetetraacetic acid-based anticoagulated tubes for laboratory analyses before CABGs. Blood samples were obtained in the morning (09.00±01.00 hours) following the 12-hour fasting period. Counts of complete and components of hemoglobin, platelets, and white blood cells (neutrophils and lymphocytes) (Sysmex K-1000 Hematology Analyzer, Guangdong, China), high-sensitivity C-reactive protein (CRP) tests, and all routine biochemical tests were performed in an autoanalyzer (Roche Diagnostic Modular Systems, Tokyo, Japan). The NLR was found by dividing the neutrophil count by the lymphocyte count, while the PLR was calculated by dividing the platelet count by the lymphocyte count. SII was calculated by multiplying the platelet count by the NLR.

### Operative Technique

Our hospital generally applies the median sternotomy method for sternotomy. During the grafting, aortic and venous cannulations were performed following general anesthesia and sternotomy. Graft vessels were prepared to be used by switching to cardiopulmonary bypass. An artery graft was preferred, and the left internal mammary artery was used for revascularization of the left anterior descending artery. The anastomosis was performed with saphenous venous grafts taken from the legs for other vessels. Systemic heparin (300 IU/kg) was administered during the operations. All patients underwent hypothermic and hyperkalemic blood cardioplegia antegrade for myocardial protection, and the procedures were performed under an average of 30°C (moderate) systemic hypothermia. During the operations, the cardiopulmonary bypass flow rate was kept constant (2.1-2.4 L/min/m^2^, mean perfusion pressure 40-90 mmHg). Blood transfusion was administered when needed (if hematocrit level < 20-25%). On the beating heart, distal anastomoses were performed within the time of the aortic cross-clamping, and the proximal anastomosis was performed after cardioplegia was resolved.

### Postoperative Atrial Fibrillation

NOAF after CABG was defined as any episode of AF > 30 seconds with or without symptoms, recorded by the monitoring system or 12-lead electrocardiogram (ECG) before discharge from the hospital. In the intensive care unit (ICU), the patients were connected to a 5-lead monitoring system as soon as possible and followed up on a 24-hour basis with the standard D2 lead. Daily ECG findings reveal whether NOAF develops in the patient. Moreover, when the practitioner suspects AF (when patients report a feeling of palpitations or discomfort in the chest), she/he immediately obtains a 12-lead ECG. Meanwhile, AF is defined as an irregular rhythm with the absence of discrete P waves on a 12-lead ECG.

### Statistical Analysis

We conducted all statistical analyses using IBM Corp. Released 2012, IBM SPSS Statistics for Windows, version 21.0, Armonk, NY:IBM Corp. software. We checked the distribution of quantitative variables with the Shapiro-Wilk test. We displayed descriptive data as mean±standard deviation and median (interquartile range [IQR]), depending on the normality of the distribution. When the variable did not fit the normal distribution, we used median and IQR. We run the independent samples *t*-test to compare normally distributed quantitative variables and the Mann-Whitney U test to compare non-normally distributed quantitative variables. We compared categorical variables using the Chi-squared test. We analyzed the effects of different variables on NOAF development using univariate analysis. For multivariate regression analysis, we generated the model with the parameters found to be significant (*P*<0.10) in univariate analysis. Finally, we computed Spearman’s coefficient to reveal the correlations between the relevant variables.

The predictive values of CRP, NLR, and SII were estimated by the areas under the receiver operating characteristic (ROC) curve. The area under the curve (AUC) values of each parameter mentioned were compared with the DeLong test in the MedCalc version 19.6.4, test version, statistics program (MedCalc Software Ltd, Ostend, Belgium)^[9]^.

## RESULTS

In our study, NOAF occurred in 80 (20.4%) of 392 patients. We discovered that 73 (91.3%) of these patients developed AF within the first 2-3 days during ICU follow-up. The mean age of those who developed NOAF was 59 years (52.5-62). Among them, 57 were males and 23 were females. The mean age of patients without NOAF was 53 years (47-62). Among them, 245 were males and 67 were females. Patients who developed NOAF were statistically significantly older (*P*=0.03). In the group of patients who developed NOAF, while hypertension (HT) history was significantly prevalent, LVEF was significantly lower (*P*=0.011 and *P*<0.001, respectively). Other baseline features and preoperative medications did not show statistically significant differences between the groups (Table 1).

**Table 1:** Demographic characteristics of the study populations.

	CABG		
	AF+	AF-	*P*-value
Variables	(n=80)	(n=312)	
Age (years)	59 (52.5-62)	53 (47-62)	**0.003**
Male gender (n, %)	57 (71.2%)	245 (78.5%)	0.152
Diabetes mellitus (n, %)	31 (38.7%)	104 (33.3%)	0.363
Hypertension (n, %)	52 (65%)	153 (49%)	**0.011**
Dyslipidemia (n, %)	12 (15%)	33 (10.5%)	0.268
COPD (n, %)	6 (7.5%)	28 (8.9%)	0.676
Smoking (n, %)	24 (30%)	98 (31.4%)	0.808
BMI (kg/m2)	27.4±5.2	28.1±4.6	0.261
Systolic blood pressure (mmHg)	123.8±15.1	122.6±12.4	0.493
Diastolic blood pressure (mmHg)	76.3±10.4	75.2±7.7	0.295
LVEF (%)	50.4±11.5	58.1±9.7	**< 0.001**
Left atrium (mm)	3.8 ±0.4	3.7 ±0.5	0.312
**Previous medications, n (%)**			
β-blocker	71	285	0.473
Angiotensin-aldosterone antagonists	62	260	0.224
Statin	76	296	0.963
**Operative and postoperative parameters**			
EuroSCORE II	4.78±0.89	4.56±0,9	0.097
Bypass time (min)	94.8±5.8	96±8.7	0.391
Cross-clamping time (min)	55.5±4.3	56.1±3.5	0.224
Number of bypass grafts	2.9±1.5	2.6±1.3	0.284
Duration of the hospitalization at the intensive care unit (days)	3.08±0.5	3.02±0.7	0.505
Extubation time (hours)	16.4±3.3	16.0±3.6	0.321
Intraoperative mortality (n, %)	-	-	-
In-hospital mortality (n, %)	3	9	0.689

AF=atrial fibrillation; BMI=body mass index; CABG=coronary artery bypass grafting; COPD=chronic obstructive pulmonary disease; EuroSCORE=European System for Cardiac Operative Risk Evaluation; LVEF=left ventricular ejection fraction

Considering the laboratory findings, the platelet count (265 [240-342] *vs.* 232 [194-255]; *P*<0.0001) and neutrophil count (7.4 [4.5-8.5] *vs.* 4.0 [3-6.5]; *P*<0.001) were significantly higher in the group who developed NOAF. Also, CRP levels were significantly higher in the group developing NOAF (4.7 [2.3-6.4] *vs.* 2.8 [1.1-4]; *P*<0.001); PLR levels were significantly higher in patients with NOAF (191 [122-241] *vs.* 126 [93-207]; *P*<0.001), and NLR levels were significantly higher (3.9 [3-5.9] *vs.* 2.6 [1.6-3.3]; *P*<0.001) in the group developing NOAF. When evaluated in terms of SII, a novel inflammation marker, we discovered SII levels to be statistically higher (1109 [720-2013] *vs.* 609 [373-754]; *P*<0.0001) in the group with NOAF (Table 2). The correlation analysis revealed that SII was well associated with CRP levels (r=0.777; *P*<0.001).

**Table 2:** Laboratory findings of the study populations.

	CABG		
	AF+	AF-	*P*-value
Number of patients	(n=80)	(n=312)	
Creatinine (mg/dl)	0.88±0.1	0.94±0.2	0.221
AST (U/L)	18.3±5.4	16.4±7.4	0.070
ALT (U/L)	20.1±4.5	20.7±10.8	0.684
Total cholesterol (mg/dl)	189.8±52.4	178.4±46.1	0.847
High-density lipoprotein cholesterol (mg/dl)	36.7±14.2	37.4±10.4	0.656
Low-density lipoprotein cholesterol (mg/dl)	126.6±44	117.3±41.9	0.088
Triglyceride (mg/dl)	140.7±66	125.2±70.3	0.082
Hemoglobin (mg/dL)	14.2±1.6	14.1±1.6	0.702
Platelets (10^3^/ µL)	265 (240-342)	232 (194-255)	**< 0.001**
WBC (10^3^/ µL)	9.6±4.9	8.7±3.9	0.121
Neutrophil (10^3^/ µL)	7.4 (4.5-8.5)	4.0 (3-6.5)	**< 0.001**
Lymphocyte (10^3^/ µL)	1.49 (1.1-1.9)	1.72 (1.1-2.3)	0.135
C-reactive protein (mg/l)	4.7 (2.3-6.4)	2.8 (1.1-4)	**< 0.001**
Neutrophil/lymphocyte ratio	3.9 (3-5.9)	2.6 (1.6-3.3)	**< 0.001**
Platelet/lymphocyte ratio	191 (122-241)	126 (93-207)	**< 0.001**
SII	1109 (720-2013)	609 (373-754)	**< 0.001**

AF=atrial fibrillation; ALT=alanine transaminase; AST=aspartate transaminase; CABG=coronary artery bypass grafting; SII=systemic immune-inflammation index; WBC=white blood cell

On the other hand, there was no significant difference between the groups in the operative and postoperative variables (Table 1). And the groups did not significantly differ in mortality and neurological complications.

In addition, we evaluated the role of various risk factors in NOAF development with the help of a multivariate analysis. We run multivariate logistic regression analysis with variables shown to be associated with NOAF development in the univariate analysis, such as age, HT, LVEF, and SII (Table 3).

**Table 3:** Univariate and multivariate logistic regression analyses of independent parameters for atrial fibrillation.

	Univariate analysis			Multivariate analysis		
	Odds ratio	95% CI	*P*-value	Odds ratio	95% CI	*P*-value
Age	1.036	1.011-1.062	0.005			
Hypertension	1.930	1.159-3.215	0.012			
LVEF	0.937	0.915-0.959	<0.001	0.922	0.897-0.947	<0.001
SII	1.001	1.001-1.001	<0.001	1.001	1.001-1.002	<0.001
CRP	1.132	1.040-1.232	0.004	1.108	0.987-1.244	0.046

CI=confidence interval; CRP=C-reactive protein; LVEF=left ventricular ejection fraction; SII=systemic immune-inflammation index

The multivariate logistic regression analysis showed that high SII level (odds ratio [OR]: 1.001, 95% confidence interval [CI]: 1.001-1.002; *P*<0.001) together with LVEF (OR: 0.922, 95% CI: 0.897-0.947; *P*<0.001) and high CRP level (OR: 1.108, 95% CI: 0.987-1.244; *P*=0.046) were independent predictors of NOAF development (Table 3).

The SII levels > 712.8 predicted NOAF with a sensitivity of 85% and a specificity of 61.2% (AUC: 0.781, 95% CI: 0.727-0.835; *P*<0.001). For NLR, a cutoff value of 2.96 predicted the development of NOAF with a sensitivity of 82.5% and specificity of 57%, and the AUC was 0.724 (95% CI: 0.669-0.780; *P*<0.001). For CRP, a cutoff value of 4 predicted the development of NOAF with a sensitivity of 57.5% and specificity of 81.7%, and the AUC was 0.634 (95% CI: 0.564-0.704; *P*<0.001).

**Fig. 1:**
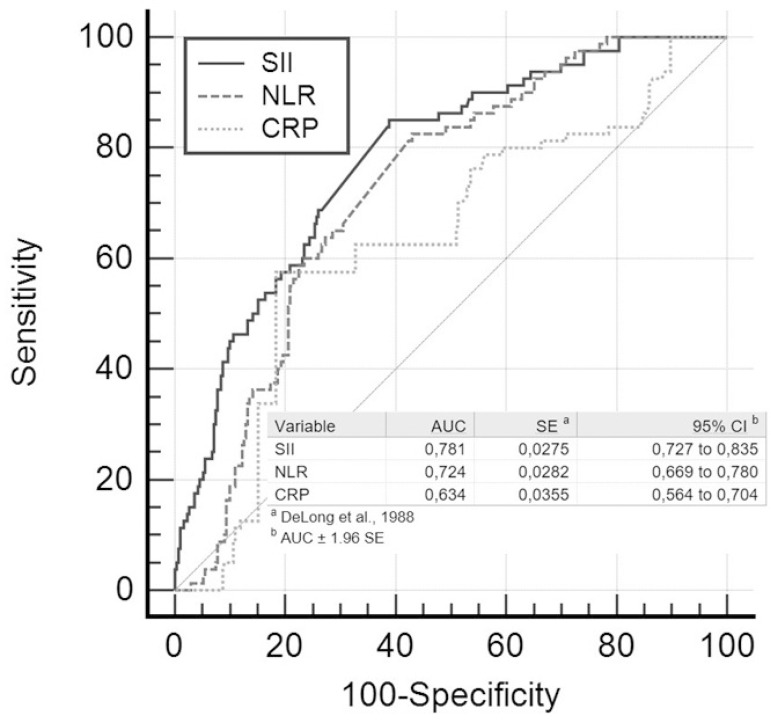
Effect of systemic immune-inflammation index (SII), neutrophil/lymphocyte ratio (NLR), and C-reactive protein (CRP) values on new-onset atrial fibrillation after coronary artery bypass grafting (receiver operating characteristic analysis). AUC=area under the curve; CI=confidence interval; SE=standard error

ROC curves were compared to assess whether SII had an additional discriminative value over serum CRP and NLR levels. We found that SII had a higher accuracy in predicting NOAF compared with serum NLR alone (SII *vs.* NLR, AUC: 0.781 *vs.* 0.724, z=4.117; *P*<0.0001). Also, the SII value had similar discriminatory power in predicting NOAF when compared with the CRP (SII *vs.* CRP, AUC: 0.781 *vs.* 0.634, z =2.824; *P*=0.0047). However, CRP and NLR had a similar accuracy for predicting NOAF (NLR *vs.* CRP, AUC: 0.724 *vs.* 0.634, z=1.732; *P*=0.0833) (Figure 1).

In addition, to better evaluate the prognostic significance of SII on NOAF, we selected 153 patients from the non-NOAF group whose characteristics were similar to the group with AF. We discovered that SII levels continued to be statistically higher than those without NOAF. (Table 4). When we re-performed the regression analysis with SII and CRP, which were shown to have an effect on the development of NOAF, high SII level (OR: 1.002, 95% CI: 1.001-1.002; *P*<0.001) as well as high CRP level (OR: 1.121, 95% CI: 1.013-1.241; *P*=0.004) were also independent predictors of NOAF development (Table 5).

**Table 4:** Demographic characteristics of the study populations (matched group from non-new-onset atrial fibrillation group).

	CABG		
	AF+	AF-	*P*-value
Variables	(n=80)	(n=153)	
Age (years)	59 (52.5-62)	57 (50-64.5)	0.990
Male gender (n, %)	57 (71.2%)	119 (77.7%)	0.271
Diabetes mellitus (n, %)	31 (38.7%)	56 (36.6%)	0.747
Hypertension (n, %)	52 (65%)	91 (59.4%)	0.411
Dyslipidemia (n, %)	12 (15%)	22 (14.3%)	0.899
COPD (n, %)	6 (7.5%)	7 (4.5%)	0.356
Smoking (n, %)	24 (30%)	52 (33.9%)	0.538
BMI (kg/m2)	27.4±5.2	27.7±4.7	0.585
LVEF (%)	50.4±11.5	51.6±8.1	0.346
Left atrium (mm)	3.8 ±0.4	3.7±0.4	0.412
**Laboratory findings of the study populations**			
WBC (10^3^/µL)	9.6±4.9	8.4±3.4	0.056
Platelets (10^3^/µL)	265 (240-342)	240 (205-258)	**< 0.001**
Neutrophil (10^3^/µL)	7.4 (4.5-8.5)	3.8 (3-5.9)	**< 0.001**
Lymphocyte (10^3^/µL)	1.49 (1.1-1.9)	1.68 (1-2.5)	0.332
C-reactive protein (mg/l)	4.7 (2.3-6.4)	3.5 (1.3-4)	**< 0.004**
Neutrophil/lymphocyte ratio	3.9 (3-5.9)	2.8 (1.6-3)	**< 0.001**
Platelet/lymphocyte ratio	191 (122-241)	144 (94-240)	**0.001**
SII	1109 (720-2013)	628 (396-720)	**< 0.001**

AF=atrial fibrillation; BMI=body mass index; CABG=coronary artery bypass grafting; COPD=chronic obstructive pulmonary disease; LVEF=left ventricular ejection fraction; SII=systemic immune-inflammation index; WBC=white blood cells

**Table 5:** Univariate and multivariate logistic regression analyses of independent parameters for atrial fibrillation.

	Univariate analysis			Multivariate analysis		
	Odds ratio	95% CI	*P*-value	Odds ratio	95% CI	*P*-value
SII	1.002	1.001-1.002	<0.001	1.002	1.001-1.002	<0.001
CRP	1.121	1.013-1.241	0.027	1.185	1.055-1.332	0.004

CI=confidence interval; CRP=C-reactive protein; SII=systemic immune-inflammation index

## DISCUSSION

In our study, we focused on the relationship between SII and NOAF development after CABG. Blood samples taken from patients developing NOAF after CABG had dramatically higher CRP, NLR, and SII levels compared to those who did not develop NOAF. The most striking finding of this study was that the SII level was the most effective predictor for patients with NOAF, among all other variables.

Postoperative NOAF is the most prevalent arrhythmic complication after the cardiac operations and is seen almost 50 times more than in the general population^[10]^. Postoperative NOAF development increases problems, such as a longer stay in an ICU, morbidity, and higher treatment costs^[11]^. Male gender, advanced age, chronic obstructive pulmonary disease, HT, and lower LVEF are the well-known risk factors for NOAF development^[12]^. Our findings are consistent with the literature, and low LVEF was found to be an independent predictor of the development of NOAF after CABG^[12]^.

Although the pathophysiological mechanisms leading to AF development are not fully and accurately revealed, previous studies proved that inflammation, increased inflammatory response, and oxidative stress play an important role in the development and progression of AF^[3,13-14]^. Gibson et al.^[15]^ and Ji et al.^[16]^ showed a relationship between NLR and CRP levels in the NOAF development in patients undergoing CABG. Gungor et al.^[5]^ proved that patients with preoperative high PLR levels are at higher risk of NOAF after CABG. In our study, our results on NLR and CRP were consistent with the results of previous studies. However, we could not find a relationship between PLR and NOAF.

It is a prevailed idea that SII can define the immune and inflammatory status in patients more comprehensively than single-component (neutrophils, lymphocytes, and platelets) and two-component (PLR and NLR) inflammatory predictors. Indeed, the results obtained from recent studies showed that high SII levels are superior to NLR and PLR in predicting the risk of adverse clinical outcomes in patients with various diseases^[7]^. With its increasing popularity in recent years, its use in the evaluation of cardiovascular diseases has gained substantial momentum.

Huang et al.^[8]^ found that increased SII levels in ST-elevation myocardial infarction patients treated with percutaneous coronary intervention are associated with both long- and short-term poor clinical outcomes. In addition, Erdoğan et al.^[17]^ found that SII elevation is an independent predictor for determining functionally significant coronary stenosis detected by fractional flow reserve in patients with chronic coronary syndrome. Our study resulted in a strong relationship between NOAF and SII after CABG. Compared to previous studies, we found that SII was the most robust and independent marker, among others, associated with NOAF development. We also found that the optimal cutoff point for SII was 712.8, which predicted NOAF development after CABG with 85% sensitivity and 61.2% specificity.

We speculate that some mechanisms mediate SII, which leads it to be the most potent predictive marker that contributes to NOAF formation compared to other markers. As it is known, increased neutrophil represents the activation of inflammation, while lymphopenia is an indicator of physiological stress. NLR shows the balance between neutrophil and lymphocyte counts and can be considered as a measure of the response to stress as well as systemic inflammation. There are studies showing that CABG is associated with neutrophil activation and may cause perioperative myocardial damage^[18]^. In addition, neutrophils can cause hypercoagulability and are associated with reperfusion injury^[19]^. It is obvious that CABG causes serious stress, and it is reasonable to cause a decrease in lymphocyte count. Increased NLR levels are associated with developing arrhythmias^[6,20,21]^. Platelets play an important role in hemostasis, which is a physiological response occurring to prevent extravasation of the blood when vascular damage happens. In addition, they have both an inflammatory effect and activate the immune system by releasing chemokines and cytokines^[22]^. Moreover, Scott et al.^[23]^ and Jalife et al.^[24]^ reported that infiltration of the myocardial tissue of the atrium by leukocytes, as well as some inflammatory cytokines released by both leukocytes and platelets, can cause AF through multiple mechanisms such as promoting atrial electrical, structural, and contractile remodeling.

Our findings are consistent with previous reports highlighting the relationship between inflammation and AF development. The hallmark of the present study was the comparison of several inflammatory markers (neutrophil, lymphocyte, NLR, PLR, and CRP) that were previously shown to be associated with the development of postoperative NOAF in patients.

### Limitations

The main limitation of this study is that we collected the data retrospectively. Cardiac rhythm was monitored only during hospitalization in the ICU. Therefore, we may have ignored the possible silent AF in the service follow-up of patients. Also, this study was a single-center study with a relatively small number of patients. We omitted CABGs performed on the beating heart in the study. Even though the operations were performed by a single surgeon, the difference between operators cannot be ignored. We could not compare the specific roles of drugs in patients with and without NOAF, as the treatment algorithm was applied to all subjects undergoing CABG and almost all subjects received the same agents for medical treatment. Another limiting factor is the evaluation of SII levels with only at admission. We also did not evaluate follow-up periods and used only a single measurement for SII. Finally, we did not include long-term follow-ups of the patients.

## CONCLUSION

In conclusion, the current study results uncovered that SII, which shows the inflammatory state as a useful, simple, easily measurable, and cheap indicator, is the most robust inflammatory marker that predicts NOAF risk in patients after CABG. More comprehensive and multicenter studies should be conducted to propose a better analysis of all possible predictors of AF.
